# Detection of the mcr-1 gene in bacteriaemia caused by *Escherichia coli* and *Klebsiella pneumoniae*

**DOI:** 10.17843/rpmesp.2024.412.13507

**Published:** 2024-05-27

**Authors:** Coralith García, Lizeth Astocondor, Noemi Hinostroza, Fiorella Krapp, Jan Jacobs

**Affiliations:** 1 Alexander von Humboldt Institute of Tropical Medicine, Universidad Peruana Cayetano Heredia. Lima, Peru. Universidad Peruana Cayetano Heredia Alexander von Humboldt Institute of Tropical Medicine Universidad Peruana Cayetano Heredia Lima Peru; 2 Cayetano Heredia National Hospital. Lima, Peru. Cayetano Heredia National Hospital Cayetano Heredia National Hospital Lima Peru; 3 Antwerp Institute of Tropical Medicine. Antwerp, Belgium Antwerp Institute of Tropical Medicine Amberes Belgium

To the Editor. Since its emergence, isolates of carbapenem-resistant *Klebsiella pneumoniae* have been disseminated in hospitals in different regions of Peru. Currently, carbapenemase type New Delhi metallo-beta-lactamase (NDM) is the most frequently detected among *K. pneumoniae* and *Escherichia coli* isolates ^1^. In these infections, colistin is often the antibiotic of last resort, since new antibiotics against these pathogens are not included in Peru’s National Drug Formulary.

In order to assess colistin susceptibility, the recommended method for estimating the minimum inhibitory concentration is broth microdilution, but this method is not practical for most microbiology laboratories. In contrast, the disk elution method uses reagents and equipment usually available in routine laboratory processes and is less time consuming. This method is also recommended by the Clinical and Laboratory Standards Institute (CLSI) for the evaluation of colistin in *Enterobacteriaceae*^2^.

The mechanisms of colistin resistance among gram-negative bacilli include mainly resistance genes located in chromosomes or in transferable elements (plasmids or transposons), the latter being the most widespread mechanism. The *mcr-*1 gene, including its variants and sub-variants that are carried on these plasmids or transposons have been detected worldwide among several species of gram-negative bacteria either in humans, animals or in the environment. The *mcr* genes encode a transferase that increases the cationic charge of the lipopolysaccharide causing decreased binding of colistin to the lipopolysaccharide ^3^.

We evaluated colistin susceptibility in 317 isolates of *E. coli* (n=199) and *K. pneumoniae* (n=118) obtained from blood cultures. These samples were collected during an antimicrobial resistance surveillance study that included 15 hospitals in 12 regions of Peru between 2017-2019 ^4^. Bacterial identification was performed through conventional biochemical tests and susceptibility to carbapenems tests were performed through the disc-diffusion method. In order to assess susceptibility to colistin, the disk elution technique was performed following the procedures described by CLSI ^2^, identifying 9.1% (29/317) isolates resistant to colistin (≥4 μg/mL), of which 7.5% (15/199) and 11.9% (14/118) were *E. coli* and *K. pneumoniae* isolates, respectively (Table 1). Regarding resistance to carbapenems, 11.0% of *K. pneumoniae* isolates were resistant to some carbapenemics, on the other hand, none of the *E. coli* isolates were found to be resistant.

We evaluated the presence of the *mcr*-1 gene by conventional PCR technique in all colistin-resistant isolates. The primers CLR-5F and CLR-5R described by Liu *et al*. ^5^^)^ were used. The master mix was prepared to a final volume of 25 μl with 0.2 μM of each primer, 0.2 mM dNTP, 1.5 mM MgCl2, 1X buffer, 0.03 U/μl of GOTAQ® G2 FLEXI DNA polymerase (Promega, Madison, USA) and 2 μl of DNA. The cycling conditions described by Faccone *et al*. were used with a modification in the hybridization temperature of 72 °C for 5 min. The size of the amplicons was 309 base pairs.

**Table 1:** Distribution of colistin resistance and *mcr*-1 gene detection among *Escherichia coli* and *Klebsiella pneumoniae* isolates in 15 hospitals in 12 regions of Peru.

Hospital	Region	*Escherichia coli*					*Klebsiella pneumoniae*				
		N	Colistin resistance		**Detection of *mcr*-1 gene**		N	Colistin resistance		**Detection of *mcr*-1 gene**	
			n	(%)	n	(%)		n	(%)	n	(%)
1	Lima	88	6	(6.8)	1	(1.1)	47	6	(12.8)	0	(0.0)
2	Lima	23	3	(13.0)	3	(13.0)	9	1	(11.1)	0	(0.0)
3	Lima	17	1	(5.9)	0	(0.0)	19	2	(10.5)	0	(0.0)
4	La Libertad	16	1	(6.3)	1	(6.3)	6	1	(16.7)	0	(0.0)
5	La Libertad	12	1	(8.3)	1	(8.3)	8	0	(0.0)	-	-
6	Cusco	9	0	(0.0)	-	-	4	0	(0.0)	-	-
7	Arequipa	8	1	(12.5)	0	(0.0)	5	0	(0.0)	-	-
8	Madre de Dios	6	1	(16.7)	1	(16.7)	2	0	(0.0)	-	-
9	Tacna	5	0	(0.0)	-	-	2	1	(50.0)	0	(0.0)
10	Loreto	4	0	(0.0)	-	-	6	3	(50.0)	1	(16.7)
11	Ancash	4	1	(25.0)	1	(25.0)	2	0	(0.0)	-	-
12	Ucayali	3	0	(0.0)	-	-	0	0	(0.0)	-	-
13	Lambayeque	2	0	(0.0)	-	-	5	0	(0.0)	-	-
14	Ica	2	0	(0.0)	-	-	1	0	(0.0)	-	-
15	Tumbes	0	0	(0.0)	-	-	2	0	(0.0)	-	-
	Total	199	15	(7.5)	8	(4.0)	118	14	(11.9)	1	(0.8)

The *mcr*-1 gene was detected in 9 of the 29 colistin-resistant isolates, 8/15 in *E. coli* isolates and 1/14 in *K. pneumoniae* isolates (Table 1). These nine isolates were susceptible to carbapenems and were distributed in 7 of the 15 hospitals in 5 of the 12 evaluated regions (Lima, La Libertad, Ancash, Loreto and Puerto Maldonado).

The epidemiology of *Enterobacteriaceae* species carrying the *mcr*-1 gene in Peru and other Latin American countries may be underestimated since colistin resistance testing is not routinely performed on all gram-negative bacteria, and in most cases, it is reserved for those bacteria that are resistant to carbapenems. Yauri *et al*. showed that 15.2% of extended-spectrum beta-lactamase-producing enterobacteria were positive for detection of the *mcr*-1 gene in a hospital in Lima ^6^. In our study, all isolates carrying the *mcr*-1 gene were susceptible to carbapenems.

In conclusion, the presence of *Enterobacteriaceae* species carrying the *mcr*-1 gene causing bloodstream infections distributed in several hospitals and regions of Peru, alerts about the dissemination of resistance to this last-resort antibiotic in our country. Other chromosome-mediated mechanisms of colistin resistance should be explored since we found that the *mcr*-1 gene was present in less than one third of colistin-resistant bacteria.
