# Supplementary material for: Detection of the mcr-1 gene in bacteriaemia caused by Escherichia coli and Klebsiella pneumoniae
Source: Rev Peru Med Exp Salud Publica. 2024 May 27;41(2):223–4. doi: 10.17843/rpmesp.2024.412.13507 (PMC11300680; doi:10.17843/rpmesp.2024.412.13507)
Supplement: Translation

# Translation: Detección del gen MCR-1 en bacteriemias causadas por *Escherichia coli* y *Klebsiella pneumoniae*

**Authors:** Coralith García, Lizeth Astocondor, Noemi Hinostroza, Fiorella Krapp, Jan Jacobs

**Affiliations:** 1 Instituto de Medicina Tropical Alexander von Humboldt, Universidad Peruana Cayetano Heredia. Lima, Perú.; 2 Hospital Nacional Cayetano Heredia. Lima, Perú.; 3 Instituto de Medicina Tropical de Amberes. Amberes, Bélgica

Sr. Editor. Desde la emergencia de *Klebsiella pneumoniae* resistente a carbapenems, estos aislamientos han ido diseminándose en los hospitales de las diferentes regiones del Perú. Actualmente, la carbapenemasa del tipo New Delhi metallo-beta-lactamasa (NDM) es la más frecuentemente detectada entre los aislamientos de *K. pneumoniae y Escherichia coli*^1^. Frente a estas infecciones, la colistina es en muchos casos el antibiótico de último recurso, ya que los nuevos antibióticos con actividad frente a estos patógenos no se encuentran en el Petitorio Nacional Único de Medicamentos del Perú.

Para evaluar la susceptibilidad a colistina, el método recomendado para estimar la concentración inhibitoria mínima es la microdilución en caldo, pero este método no es práctico para la mayoría de los laboratorios de microbiología. En cambio, el método de elusión en disco utiliza reactivos y equipos usualmente disponibles en los procesos de rutina de los laboratorios y además consume menos tiempo. Este método además es el recomendado por el Instituto de Normas Clínicas y de Laboratorio (CLSI, por sus siglas en inglés) para la evaluación de la colistina en *Enterobacterales*^2^.

Los mecanismos de resistencia a colistina entre los bacilos gram negativos incluyen principalmente genes de resistencia localizados en cromosomas o en elementos transferibles (plásmidos o transposones), siendo este último el mecanismo más diseminado. El gen *mcr*-1, incluyendo sus variantes y sub-variantes que son transportados en estos plásmidos o transposones han sido detectados en el mundo entre varias especies de bacterias gram negativas ya sea en humanos, animales o en el medio ambiente. Los genes *mcr* codifican una transferasa que incrementa la carga catiónica del lipopolisacárido causando la disminución de la unión de la colistina con el lipopolisacárido ^3^.

Evaluamos la susceptibilidad a colistina en 317 aislamientos de *E. coli* (n=199) y *K. pneumoniae* (n=118) obtenidos de hemocultivos. Esta recolección se realizó a través de un estudio de vigilancia de la resistencia antimicrobiana que incluyó 15 hospitales en 12 regiones del Perú entre los años 2017-2019 ^4^. La identificación de las bacterias se realizó a través de pruebas bioquímicas convencionales y la susceptibilidad a los carbapenems se realizó a través del método de disco-difusión. Para la evaluación de la susceptibilidad a colistina se realizó la técnica de elusión en disco siguiendo los procedimientos descritos por el CLSI ^2^, identificándose 9,1% (29/317) aislamientos resistentes a colistina (≥4 μg/mL) siendo 7,5% (15/199) y 11,9% (14/118) aislamientos de *E. coli* y *K. pneumoniae*, respectivamente (Tabla 1). En relación a la resistencia a los carbapenems, el 11,0% de aislamientos de *K. pneumoniae* fueron resistentes a algún carbapenémico mientras que en el caso de *E. coli* no se encontró ningún aislamiento resistente.

**Tabla 1:** Distribución de la resistencia a colistina y la detección del gen mcr-1 entre aislamientos de *Escherichia coli* y *Klebsiella pneumoniae* en 15 hospitales de 12 regiones del Perú

Hospital	Región	*Escherichia coli*					*Klebsiella pneumoniae*				
		N	Resistencia a colistina		**Detección de gen *mcr*-1* ***		N	Resistencia a colistina		**Detección de gen *mcr*-1**	
			n	(%)	n	(%)		n	(%)	n	(%)
1	Lima	88	6	(6,8)	1	(1,1)	47	6	(12,8)	0	(0,0)
2	Lima	23	3	(13,0)	3	(13,0)	9	1	(11,1)	0	(0,0)
3	Lima	17	1	(5,9)	0	(0,0)	19	2	(10,5)	0	(0,0)
4	La Libertad	16	1	(6,3)	1	(6,3)	6	1	(16,7)	0	(0,0)
5	La Libertad	12	1	(8,3)	1	(8,3)	8	0	(0,0)	-	-
6	Cusco	9	0	(0,0)	-	-	4	0	(0,0)	-	-
7	Arequipa	8	1	(12,5)	0	(0,0)	5	0	(0,0)	-	-
8	Madre de Dios	6	1	(16,7)	1	(16,7)	2	0	(0,0)	-	-
9	Tacna	5	0	(0,0)	-	-	2	1	(50,0)	0	(0,0)
10	Loreto	4	0	(0,0)	-	-	6	3	(50,0)	1	(16,7)
11	Ancash	4	1	(25,0)	1	(25,0)	2	0	(0,0)	-	-
12	Ucayali	3	0	(0,0)	-	-	0	0	(0,0)	-	-
13	Lambayeque	2	0	(0,0)	-	-	5	0	(0,0)	-	-
14	Ica	2	0	(0,0)	-	-	1	0	(0,0)	-	-
15	Tumbes	0	0	(0,0)	-	-	2	0	(0,0)	-	-
	Total	199	15	(7,5)	8	(4,0)	118	14	(11,9)	1	(0,8)

En todos los aislamientos resistentes a colistina, evaluamos la presencia del gen *mcr*-1 a través de la técnica de PCR convencional. Se utilizaron los *primers* CLR-5F y CLR-5R descritos por Liu *et al*. ^5^. Se preparó el master mix para un volumen final de 25 μl con 0,2 μM de cada primer, 0,2 mM de dNTP, 1,5 mM de MgCl2, 1X de buffer, 0,03 U/μl de GOTAQ® G2 FLEXI DNA polimerasa (Promega, Madison, USA) y 2 μl de ADN. Se usaron las condiciones de ciclaje descritas por Faccone *et al*. con una modificación en la temperatura de hibridación de 72 °C por 5 minutos. El tamaño de los amplicones fue de 309 pares de bases.

El gen *mcr*-1 fue detectado en 9 de los 29 aislamientos resistentes a colistina, 8/15 en aislamientos de *E. coli* y 1/14 de *K*. *pneumoniae* (Tabla 1). Estos nueve aislamientos fueron susceptibles a *carbapenems* y estuvieron distribuidos en 7 de los 15 hospitales de 5 de las 12 regiones evaluadas (Lima, La Libertad, Ancash, Loreto y Puerto Maldonado).

La epidemiologia de las especies de *Enterobacterales* portadores del gen *mcr*-1 en el Perú y otros países de Latinoamérica puede ser subestimada dado que el análisis de la resistencia a colistina no es realizado de rutina a todas las bacterias gram negativas, ya que en la mayoría de casos se reserva a aquellas bacterias que son resistentes a carbapenems. Yauri *et al*. mostraron que 15,2% de enterobacterias productoras de betalactamasas de espectro extendido fueron positivas para la detección del gen *mcr*-1 en un hospital de Lima ^6^. En nuestro estudio, todos los aislamientos portadores del gen *mcr*-1 fueron susceptibles a carbapenems.

En conclusión, la presencia de especies de *Enterobacterales* portadoras del gen *mcr-*1 causando infecciones del torrente sanguíneo distribuidas en varios hospitales y regiones del Perú, alerta sobre la diseminación de resistencia a este antibiótico de último recurso en nuestro país. Otros mecanismos de resistencia a colistina mediados por cromosomas deberían ser explorados dado que encontramos que el gen *mcr*-1 estuvo presente en menos de un tercio de las bacterias resistentes a colistina.
